# Childhood maltreatment preceding depressive disorder at age 18 years: A prospective Brazilian birth cohort study

**DOI:** 10.1016/j.jad.2017.03.065

**Published:** 2017-08-01

**Authors:** Erika Alejandra Giraldo Gallo, Christian Loret De Mola, Fernando Wehrmeister, Helen Gonçalves, Christian Kieling, Joseph Murray

**Affiliations:** a Postgraduate Program in Epidemiology, Universidade Federal de Pelotas, Rua Marechal Deodoro, 1160-3° Piso. Pelotas RS, Brazil; b Graduate School of Nursing, Universidade Federal de Pelotas, Brazil; c Department of Psychiatry, School of Medicine, Hospital de Clínicas de Porto Alegre, Universidade Federal do Rio Grande do Sul, Brazil; d Department of Psychiatry, University of Cambridge, England

**Keywords:** Childhood maltreatment, Abuse, Neglect, Depression, Birth cohort, Middle-income country

## Abstract

**Background:**

Childhood maltreatment is linked with increased risk for mental illness in adolescence and adulthood. However, little evidence is available on whether different forms of maltreatment have specific effects, and no prospective studies in low- or middle-income countries have addressed this issue.

**Methods:**

Participants in a population-based, birth cohort study in Pelotas, Brazil (N=3715) self-reported exposure to maltreatment (emotional abuse, physical neglect, physical abuse, sexual abuse, domestic violence) in confidential questionnaires at age 15 years, and were assessed for major depression in interviews at age 18 years, using the MINI. Confounding variables concerning family characteristics were measured in interviews with mothers in the perinatal period and at age 11 years.

**Results:**

Females exposed to emotional abuse (OR=2.7; 95%CI=1.9, 3.8) and domestic violence (OR=1.9; 95%CI=1.2, 2.9) were at increased risk for depression after adjustment for confounders and other types of maltreatment. Females exposed to two or more forms of maltreatment were at particularly high risk for depression (OR=4.1; 95%Cl=2.8, 6.1) compared with females not exposed to maltreatment. In adjusted analyses, maltreatment was not associated with depression for males.

**Limitations:**

Detailed information about maltreatment such as timing and frequency was not available, and 1534 individuals were not included in the analyses, who had poorer and less educated mothers.

**Conclusions:**

Emotional abuse and domestic violence are strong risk factors for major depression for females. Early intervention to prevent maltreatment and its consequences is critical, especially for girls exposed to poly-maltreatment.

## Highlights

• Little is known about effects of different forms of child maltreatment on depression.
• We conducted a large, population-based birth cohort study in Brazil.
• Emotional abuse and domestic violence increased risk for depression at age 18 for females.
• Exposure to multiple forms of maltreatment was a particularly strong risk factor for females.
• No effects of maltreatment on depression were found for males, controlling for other variables.

## 1 Introduction

Worldwide, 23% of adults report having been physically abused as a child, 36% report having suffered emotional abuse, and 18% of women and 8% of men report experiences of child sexual abuse (Stoltenborgh et al., 2015). Child maltreatment refers to physical, sexual and emotional violence, and neglect of children and adolescents by parents, caregivers and other authority figures (World Health Organization, 2016). Exposure to intimate partner violence is also sometimes included as a form of child maltreatment. This broad range of childhood maltreatment is associated with increased risk of mental illness, including depression, post-traumatic stress disorder, and anxiety disorders (Li et al., 2016). However, empirical evidence is sparse on the specificity of effects of different forms of maltreatment on mental illness (Alloy et al., 2006, Infurna et al., 2016). It is hypothesised that the effects of different forms of abuse and neglect vary, with emotional abuse having particularly strong associations with depression, because of its effects on cognitive schemas about worthlessness and loss, and physical and sexual abuse having stronger effects on anxiety, given heightened perception of threats and danger (Alloy et al., 2006; Kaplan et al.; Lumley and Harkness, 2007; Shapero et al., 2014).

Depression is a major public health problem. Major depressive disorder was responsible for the highest proportion (24.5%) of Disability-Adjusted Life Years caused by mental, neurological, and substance use disorders in 2010 (Whiteford et al., 2015). Stressful life events have been consistently associated with increased risk of depression (Colman and Ataullahjan, 2010), and it is estimated that as many as half of the cases of depression and anxiety worldwide may be attributable to childhood maltreatment (Li et al., 2016). Women are about twice as likely as men to suffer from depression, and an important cause of this sex difference might be the higher rate of child sexual abuse among girls compared with boys (Cutler and Nolen-Hoeksema, 1991, Stoltenborgh et al., 2015).

From a life-course perspective, several studies have linked adult mental illness with exposure to risk factors during critical periods of development during gestation, early childhood, late childhood and adolescence, as well as cumulatively through the life course (Ben-Shlomo and Kuh, 2002, Schlotz and Phillips, 2009). Given known biases in adult retrospective measures of childhood maltreatment (Hardt and Rutter, 2004), prospective studies are particularly important for elucidating consequences of maltreatment through time. In a recent systematic review, five prospective studies were found that showed a positive association between general child maltreatment and adult depression, with a pooled odds ratio (OR) of 2.0 (Li et al., 2016). In the same review, a combined outcome of depression and anxiety was examined in relation to specific types of maltreatment, producing pooled odds ratios of 2.0 for physical abuse, 2.7 for sexual abuse and 1.7 for neglect; however, specific effects of each type of maltreatment on depression were not reviewed. Another recent systematic review examined depression as a specific outcome of different forms of maltreatment, but given the dearth of longitudinal studies, cross-sectional and case-control studies had to be included as well as prospective studies (Infurna et al., 2016). The overall effect size (d) for the association with depression across all these studies was 0.93 for emotional abuse, 0.81 for physical abuse, 0.50 for sexual abuse, and 0.81 for neglect. Ideally, new prospective studies would be conducted to elucidate whether specific forms of maltreatment have different effects on depression.

Notably, all studies of maltreatment and depression identified in two recent systematic reviews (Infurna et al., 2016, Li et al., 2016) were conducted in high-income countries. Ribeiro et al. (2009) found no prospective studies of maltreatment and depression in LMICs. About 90% of children and youth (aged 0–29 years) live in LMICs (United Nations Department of Economic and Social Affairs Population Division, 2015), where there are fewer social support services for children and fewer psychological and psychiatric treatment resources than in high-income countries, which may amplify the risks associated with maltreatment for children. One recent study in Pelotas, Brazil found that general maltreatment predicted depression in late adolescence, with an amplification in that effect among individuals possessing a small 5-HTTLPR allele (Rocha et al., 2015). Brazil is a middle-income country with a high-rate of violence (Murray et al., 2013, Reichenheim et al., 2011), and one of the highest rates of child maltreatment worldwide, according to the Childhood Trauma Questionnaire (Viola et al., 2016). The current study aimed to investigate prospective associations between specific forms of childhood maltreatment and depression in late adolescence in a Brazilian birth cohort.

## 2 Methods

### 2.1 The 1993 Pelotas birth cohort study

Pelotas city is situated in the extreme south of Brazil in Rio Grande do Sul state, with an estimated population of 343,000 inhabitants (IBGE, 2015). The 1993 Pelotas birth cohort is a population-based study designed to assess the trends in maternal and child health indicators, and evaluate to the associations between early life variables and later health outcomes. All births occurring between 1st February and 31st December of 1993 in the five maternity clinics in the town were monitored (99% of births in Pelotas occurred in hospital). For the 5265 children born alive, only 16 mothers could not be interviewed or refused to participate in the study, and 5249 (99.6%) were included in the initial cohort. The mothers were interviewed about demographic, socioeconomic, and health-related characteristics and the children have been followed in ongoing assessments. In 2004/2005, 2008/2009 and 2011/2012, all the cohort members were sought at ages 11, 15 and 18 years, with response rates of 87.5%, 85.7% and 81.4%, respectively. At ages 11 and 15, mothers as well as cohort members were interviewed, but at age 18 only cohort members were interviewed. More details on the methodology can be found in other publications (Gonçalves et al., 2014, Victora et al., 2008).

**Fig. 1:**
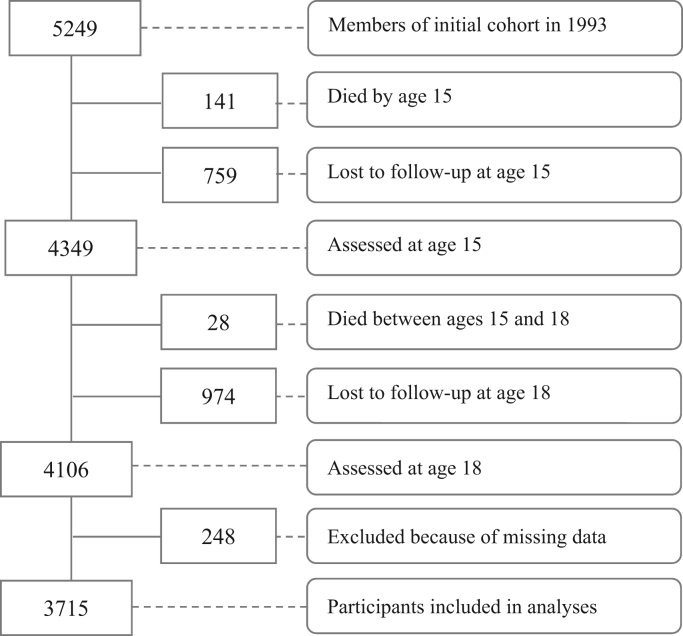
Flow-chart of participant inclusion in current study and analyses: Pelotas (Brazil) Birth Cohort.

For the current analyses, 3715 (70.8% of the original cohort) participants were included who had data on both depression at age 18 years and complete data on maltreatment at age 15 years. Participants were excluded from the current analyses if they had died or were lost to follow-up by age 18, if they had missing data on maltreatment at age 15, or missing data on depression at age 18 (see Fig. 1).

The study protocol and all follow-ups were approved by the Medical Ethics Committee of the Federal University of Pelotas. Participating mothers gave prior and informed consent. At age 18 young members signed their own consent form, agreeing to participate in the research.

### 2.2 Measures

#### 2.2.1 Major depression

Major depression was assessed for the first time in the age 18 visit (2011–2012). All cohort members were assessed by trained psychologists for psychiatric diagnoses, using the Mini International Neuropsychiatric Interview (MINI V5.0) (Sheehan et al., 1998), which has been validated for use in Brazil (Amorim, 2000). The MINI is a standardized diagnostic interview that assesses the major psychiatric disorders of axis I according to the Diagnostic and Statistical Manual of Mental Disorders – IV revision (DSM-IV) and the International Statistical Classification of Diseases and Related Health Problems – 10th Revision (ICD-10). This study considered as current major depression cases individuals who scored positive for an episode of depression during the past two weeks.

#### 2.2.2 Maltreatment and domestic violence

Information about exposure to maltreatment was obtained at the age 15 home visit (2008). In this large, population-based study a long interview to determine maltreatment was not possible. Selected items were adapted from the Brazilian version of the Childhood Trauma Questionnaire (Grassi-Oliveira et al., 2006), to measure indicators of physical neglect, physical abuse, emotional abuse, sexual abuse, as well as domestic violence. Participants completed a confidential questionnaire which was applied in a private place and placed in a sealed envelope after completion. Following prior work with this questionnaire (Soares et al., 2016), our exposures were defined according to positive answers to the following questions:

**Physical neglect:** Have you ever not had enough food at home or had to wear dirty or torn clothes because you had no others?
**Physical abuse:** Has an adult in your family or someone who was looking after you hit you in a way that left you hurt or bruised?
**Emotional abuse:** Have you ever thought or felt that your parents did not want you to have been born? OR Have you ever thought or felt that someone in your family hates you?
**Sexual abuse:** Has anyone ever tried to do sexual things to you against your will, threatening or hurting you?
**Domestic violence:** Has there ever been fights with physical assault in your household between adults or has an adult ever assaulted a child or adolescent?
**Number of types of maltreatment:** This variable was constructed by counting the number of types of maltreatment above (out of 5), and was categorised as exposure to 0, 1, or 2 or more types of maltreatment.

### 2.3 Covariates

Variables used to adjust for confounding in multivariate models were selected from assessments during the perinatal period and age 11 years. Covariates used as confounds in this study were selected empirically, on the basis that they were associated with both maltreatment and depression. Only variables associated (p<0.20) with at least one form of maltreatment as well as depression were included as confounding variables. Factors included as confounding variables from the perinatal interview in 1993 were: maternal age (≤19/≥20 years), skin colour self-reported by the mother (white/non-white); smoking during pregnancy (yes/no); parity (0–1/2–3/≥4 children); mother's marital status (no partner/with partner); mother's schooling, referring to the number of complete years of study (0–4: low/5–8: medium/≥ 9 years: high); quintiles of household income, and child sex (male/female). From the age 11 assessment in 2004, classification of the mental health of the child's mother was adjusted for in multivariate models. Mothers answered the Self-Reporting Questionnaire (SRQ-20) adapted and validated in Brazil (Mari and Williams, 1986) and were classified as having a probable mental health disorder if the total score was greater than or equal to eight points.

### 2.4 Statistical analyses

To assess the association between maltreatment experiences and risk for depression, logistic regression was used to calculate odds ratios (ORs) with 95% confidence intervals (95% CIs), separately for each type of maltreatment. Analyses were stratified by child sex, because of sex differences in rates of maltreatment and depression, and because tests of interaction showed that some effects of maltreatment on depression differed between females and males. We estimated a multivariate model for each type of maltreatment exposure, which adjusted for all other forms of maltreatment and all confounders from the perinatal period and maternal depression measured at 11 years. All analyses were conducted using Stata, version 10.0. (Stata inc., Texas, USA).

## 3 Results

Of the 3715 individuals included in the current study, 52.6% were female. The mothers of the included participants had the following characteristics: 17.0% were teenage mothers, 76.8% had white skin colour, 33.1% smoked during pregnancy, 63.7% were primiparous, 11.9% were without husband or partner, 26.3% had a low level of education, and 18.9% were in the lowest income quintile (of the whole cohort). Table 1 compares characteristics of the participants included in analyses with participants who were excluded because of missing data or loss to follow-up by age 18 years. Included participants were significantly (p<0.050) more likely to be female, and mothers of included participants were slightly less likely to have a low educational level and to have a low family income.

**Table 1:** Participant sociodemographic and maternal characteristics according to their inclusion/exclusion in the current analyses. The 1993 Pelotas (Brazil) birth cohort.

**Sociodemographic and maternal characteristics**	**Total**	**Included in current analyses**	**Excluded from current analyses**	
	**N=5249**	**N=3715**	**N=1534**	
	***%***	***%***	***%***	***p*****value**
*Perinatal assessment*				
Sex				<0.001
Female	50.3	52.6	44.9	
Male	49.7	47.4	55.1	
Maternal age				0.245
≤19	17.4	17.0	18.4	
≥20	82.6	83.0	81.6	
Maternal skin colour				0.177
White	77.3	76.8	78.6	
Non-white	22.7	23.2	21.4	
Smoking during pregnancy				0.520
No	66.6	66.9	66.0	
Yes	33.4	33.1	34.0	
Maternal parity				0.123
0–1 siblings	62.9	63.7	60.8	
2–3 siblings	25.8	25.2	27.1	
≥4 siblings	11.4	11.1	12.1	
Mother lives with a partner				0.110
No	12.4	11.9	13.5	
Yes	87.6	88.1	86.5	
Maternal education				<0.001
Low	28.0	26.3	32.1	
Medium	46.2	48.3	41.2	
High	25.8	25.4	26.7	
Family income in quintiles				0.018
1st (lowest)	20.1	18.9	22.8	
2nd	23.3	23.4	22.9	
3rd	17.3	17.7	16.4	
4th	19.5	20.2	17.9	
5th (wealthiest)	19.8	19.8	20.0	

*11 year follow-up assessment*				
Mother common mental disorders				0.124
No	59.9	60.4	57.4	
Yes	40.1	39.6	42.6	

Notes. *p* value from Chi-squared test for the difference between included and excluded participants.

The overall prevalence of major depression was 6.8%, with a higher rate (10.0%) for women than for men (3.3%) (p<0.001 for the difference by sex). Table 2 shows the risk of major depression by socioeconomic and demographic characteristics of participants, separately for females and males. For both sexes, major depression was associated with maternal smoking in pregnancy, maternal low education, and low family income. For females only, depression was also associated with having a young mother at birth; for males only, depression was also associated with maternal non-white skin colour and maternal mental health problems (all p<0.050).

**Table 2:** Prevalence of depression at age 18 years according to sociodemographic and maternal characteristics. The 1993 Pelotas (Brazil) birth cohort.

**Sociodemographic and maternal characteristics**	**Women**		**Men**	
	**N**	**% Depressed**	**N**	**% Depressed**
*Perinatal assessment*				
Maternal age		*p=0.005*		*p=0.059*
≤19	313	14.4	320	5.0
≥20	1641	9.1	1440	2.9
Maternal skin colour		*p=0.108*		*p=0.030*
White	1492	9.4	1361	2.8
Others	460	12.0	400	5.0
Smoking during pregnancy		*p<0.001*		*p=0.008*
No	1292	8.0	1193	2.7
Yes	662	13.9	568	5.0
Maternal parity		p=0.063*		p=0.938*
0–1 siblings	1234	9.2	1134	3.4
2–3 siblings	515	10.7	422	2.6
≥4 siblings	205	13.2	205	3.9
Mother lives with a partner in 1993		*p=0.142*		*p=0.062*
No	219	12.8	223	5.4
Yes	1735	9.6	1538	3.0
Maternal education		p<0.001*		p=0.003*
Low	521	12.5	454	5.5
Medium	934	11.2	859	2.8
High	496	4.8	445	2.0
Family income (quintiles)		p=0.001*		p=0.031*
1st (poorest)	372	12.1	320	3.8
2nd	429	13.5	426	4.7
3rd	340	10.3	305	3.9
4th	385	6.0	351	1.7
5th (richest)	391	7.7	333	2.1

*11 year follow-up assessment*				
Mother common mental disorders		*p=0.334*		*p=0.004*
No	1182	9.4	1017	2.3
Yes	735	10.8	708	4.8
**Total**	**1954**	**10.0**	**1761**	**3.3**

Notes. Row percents; *p* value from Chi-squared test.

* *p* value from trend analysis.

Table 3 shows the number of participants exposed to each type of maltreatment, and rates of major depression according to maltreatment exposure**,** separately for females and males. Females were more likely to be exposed to all forms of maltreatment than males (all p<0.001), except for physical neglect. For women, all forms of maltreatment were significantly associated (p<0.001) with higher rates of major depression, except sexual abuse. For men, only domestic violence was significantly (p=0.030) associated with increased risk of major depression. However, for both sexes, exposure to multiple forms of maltreatment (two or more types of maltreatment) increased risk of major depression (p<0.050).

**Table 3:** Percent of participants presenting a major depressive episode at age 18 years according to types of maltreatment up to age 15 years. The 1993 Pelotas (Brazil) birth cohort.

**Maltreatment exposure**	**Women**		**Men**	
	**N**	**% Depressed**	**N**	**% Depressed**
Physical neglect		*p<0.001*		*p=0.066*
No	1876	9,4	1671	3.1
Yes	78	23,1	90	6.7
Physical abuse		*p<0.001*		*p=0.125*
No	1801	9.3	1660	3.1
Yes	153	18.3	101	5.9
Emotional abuse		*p<0.001*		*p=0.138*
No	1430	6.6	1539	3.1
Yes	524	19.3	222	5.0
Sexual abuse		*p=0.413*		*p=0.579*
No	1910	9.9	1752	3.1
Yes	44	13.6	9	0.0
Domestic violence		*p<0.001*		*p=0.030*
No	1711	8.5	1621	3.0
Yes	243	20.6	140	6.4
Number of types of maltreatment		p<0.001*		p=0.014*
None	1273	4.9	1364	2.9
1 type	436	12.2	273	3.7
2+ types	245	18.8	124	7.3
**Total**	**1954**	**10.0**	**1761**	**3.3**

Notes. N=Number of observations; *p* value Chi-squared test.

* *p* value by trend analysis.

Table 4 shows the unadjusted and adjusted associations between maltreatment exposure and major depression for females and males separately. Fully adjusted models include adjustment for confounding variables and the co-occurrence of different types of maltreatment. For females, fully adjusted models show that emotional abuse (OR=2.7; 95%CI=1.9, 3.8) and domestic violence (OR=1.9; 95%CI=1.2, 2.9) were associated with increased risk for depression. For males, no individual type of maltreatment was significantly associated with depression in adjusted models. Although there is a significant association between each type of maltreatment (Soares et al., 2016), this did not cause problems of multicollinearity (all VIF factors were <3.0 for both females and males) in the fully adjusted models. Tests of interaction between maltreatment exposure and child sex in predicting risk for major depression were performed for all five exposure variables in models including all covariates, and considered significant if p<0.200). There was no evidence for an interaction for physical neglect, physical abuse, sexual abuse, or domestic violence (all p values for tests of interaction >0.600). However, for emotional abuse, the association with depression was stronger for women than for men (test of interaction p=0.129).

**Table 4:** Odds Ratio (OR) for depression at age 18 according to maltreatment exposure up to age 15 years in the 1993 Pelotas (Brazil) Birth Cohort.

**Maltreatment Exposure**	**Women**			**Men**		
	Unadjusted	Adjusted for Confounders	Adjusted for Confounders & Maltreatment co-occurrence	Unadjusted	Adjusted for Confounders	Adjusted for Confounders & Maltreatment co-occurrence
	OR (95% CI)	OR (95% CI)	OR (95% CI)	OR (95% CI)	OR (95% CI)	OR (95% CI)
**Physical neglect**	*p<0.001*	*p=0.004*	*p*=0.189	*p=0.073*	*p=0.385*	*p=0.571*
No	1.0	1.0	1.0	1.0	1.0	1.0
Yes	2.9 (1.7, 5.0)	2.3 (1.3, 4.1)	1.5 (0.8, 2.7)	2.2 (0.9, 5.3)	1.5 (0.6, 3.7)	1.3 (0.5, 3.4)
**Physical abuse**	*p<0.001*	*p=0.007*	*p=0.683*	*p=0.132*	*p=0.210*	*p=0.659*
No	1.0	1.0	1.0	1.0	1.0	1.0
Yes	2.2 (1.4, 3.4)	1.9 (1.2, 3.0)	0.9 (0.5, 1.5)	2.0 (0.8, 4.7)	1.8 (0.7, 4.4)	1.3 (0.5, 3.4)
**Emotional maltreatment**	*p<0.001*	*p<0.001*	*p<0.001*	*p=0.142*	*p=0.210*	*p=0.462*
No	1.0	1.0	1.0	1.0	1.0	1.0
Yes	3.4 (2.5, 4.6)	3.1 (2.3, 4.3)	2.7 (1.9, 3.8)	1.7 (0.8, 3.2)	1.6 (0.8, 3.1)	1.3 (0.6, 2.8)
**Sexual abuse**	*p=0.416*	*p=0.631*	*p=0.408*			
No	1.0	1.0	1.0	NA	NA	NA
Yes	1.4 (0.6, 3.4)	1.2 (0.5, 3.0)	0.7 (0.3, 1.7)			
**Domestic violence**	*p<0.001*	*p<0.001*	*p<0.003*	*p=0.035*	*p=0.087*	*p=0.222*
No	1.0	1.0	1.0	1.0	1.0	1.0
Yes	2.8 (2.0, 4.0)	2.6 (1.8, 3.8)	1.9 (1.2, 2.9)	2.2 (1.1, 4.6)	1.9 (0.9, 4.1)	1.6 (0.7, 3.7)
	N=1954	N=1876	N=1876	N=1761	N=1697	N=1697

Notes. NA=Not applicable because no male sexual abuse victim was depressed; *p* value logistic regression; *p value from trend analysis; Confounders included in adjusted models: maternal age, maternal skin colour, smoking during pregnancy, parity, mother lives with a partner, maternal education, quintiles of family income (all measured in the perinatal period), and mother common mental disorder (when participant was 11 years old).

**Fig. 2:**
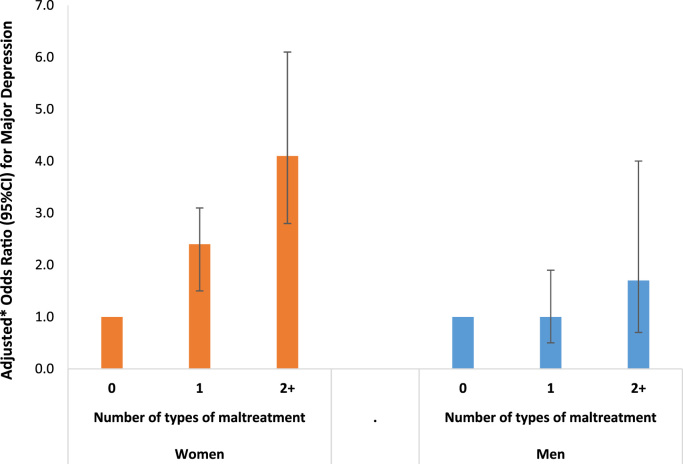
Association between the number of types of maltreatment up to age 15 and major depression at age 18 in the 1993 Pelotas Birth Cohort. * Variables adjusted for: maternal age, maternal skin colour, smoking during pregnancy, parity, mother lives with a partner, maternal education, quintiles of family income (all measured in the perinatal period), and mother common mental disorder (when participant was 11 years old).

Fig. 2 shows the association between the number of types of maltreatment and depression, adjusting for confounding variables, separately for females and males. For women, there was a strong association (p<0.001 for trend test): women who had experienced multiple types of maltreatment had 4.1 (95%CI=2.8, 6.1) times the odds of depression, compared with women exposed to no maltreatment. For males, there was not strong evidence for an association between the number of types of maltreatment exposure and depression (p=0.183 for trend test), although males exposed to multiple forms of maltreatment had twice the odds of depression (OR=2.1; 95%CI=0.9, 4.5) compared with men not exposed to maltreatment. Overall, the association between the number of types of maltreatment and depression was stronger for women than for men (p=0.183 in test of interaction).

## 4 Discussion

In a large birth cohort in Brazil, we examined multiple forms of maltreatment and domestic violence up to age 15 years in relation to major depression at age 18. The key finding was that emotional abuse and exposure to domestic violence predicted increased risk for major depression for females. These associations were independent of numerous family and individual characteristics at birth, maternal health at age 11 years, and co-occurrence with other forms of abuse/neglect. Notably, girls exposed to multiple forms of maltreatment had cumulatively higher risk for major depression. However, for males, there was no significant association between depression and any form of maltreatment in adjusted models.

Prior longitudinal research shows general maltreatment is a risk factor for major depression (Li et al., 2016), but few studies have examined the consequences of specific types of abuse and neglect, taking into account their co-occurrence (Alloy et al., 2006). In the current study, emotional abuse and exposure to domestic violence predicted depression for females, even after taking into account their overlap with other forms of maltreatment, and other family characteristics. The relatively strong effect of emotional abuse in the current study is consistent with other studies that have adjusted for the overlap between different types of child maltreatment (Gibb et al., 2001, Shapero et al., 2014). In their review, Alloy et al. (2006) concluded that, the consistent association between emotional abuse and depression contrasts with findings for physical and sexual abuse, which have been less consistent and weaker.

The current study also highlights the importance of domestic violence as a risk factor for depression for females. The increased risk found in the current study is consistent with a large literature on the detrimental consequences of domestic violence for children and adolescents (Holt et al., 2008). A meta-analysis of 58 studies of 7602 individuals, showed an overall effect size (d) of 0.48 between exposure to domestic violence and internalising problems, but nearly all studies were cross-sectional, and only about one-third were based on community samples (Evans et al., 2008). It was not clear how many of these studies adjusted for the overlap between exposure to domestic violence and other forms of child maltreatment, although common risk factors mean that they often co-occur (Guedes et al., 2016). Hence, the current, prospective, population-based study is important in demonstrating the risk of depression following exposure to domestic violence for females, even after accounting for confounders and co-occurrence with various forms of maltreatment.

The increased risk for major depression for women following multiple types of maltreatment was striking in the current study. Girls exposed to two or more types of maltreatment had 4.1 times the odds of major depression compared to girls not exposed, adjusting for confounding variables. Similarly, other studies have also found levels of depression in females increase significantly following poly-maltreatment (Cisler et al., 2012, Dong et al., 2013, du Plessis et al., 2015; Finkelhor et al., 2009; Richmond et al., 2009; Sundermann et al., 2013; Turner et al., 2006). Hence, although different forms of maltreatment often co-exist, it appears they can make unique additional contributions towards increased risk for depression. This conclusion supports a cumulative effects theory regarding increased risk for mental health problems following child maltreatment (Cicchetti and Carlson, 1989, Hooven et al., 2012). The findings also suggest the importance of identifying girls who have experienced multiple types of victimization for priority attention by mental health and social services.

There were two significant sex differences in the association between maltreatment and depression in the current study. Effects of both emotional abuse and poly-maltreatment were stronger for females than for males. Several prior studies have also found stronger associations between maltreatment experiences and mental health problems for females than males (Cutler and Nolen-Hoeksema, 1991, Thompson et al., 2004, Turner et al., 2006). Some researchers have speculated that females and males react differently to stress (Gershon et al., 2008, Nolen-Hoeksema and Girgus, 1994), with females being more likely to develop internalising symptoms (depression among others mental disorders) while males are more likely to externalise their reactions to stress (with, e.g., aggressive behaviours). For example, domestic violence has been found to have stronger associations with externalizing problems for boys than girls (Holt et al., 2008). Some theories suggest that girls are more likely than boys to blame themselves for experiencing negative life events, including maltreatment, and self-blame causes low self-esteem and increased risk for depression (Cutler and Nolen-Hoeksema, 1991). Gender inequalities and a macho culture perpetuating gendered violence in Brazil (Cecchetto et al., 2016) means that girls previously exposed to maltreatment may be particularly vulnerable to developing future depression. Other possible explanations for sex differences in the association between maltreatment and depression in the current study concern statistical power and selective attrition. The prevalence of depression among males (3.3%) was lower than among females (10.0%), and maltreatment was also less common among males than females. Hence, statistical power to detect significant associations was lower for males than females. Also, males were less likely to have complete data for inclusion in the analyses; possibly males who were exposed to maltreatment were less likely to participate, which could have biased the results.

Various possible mechanisms might link emotional abuse and domestic violence with increased risk for depression among girls. First, both types of maltreatment might cause heightened stress reactivity to later life events, causing victims to be more vulnerable to depression (Shapero et al., 2014). In particular, a negative cognitive style may mediate the link between emotional abuse and later depression (Gibb et al., 2001). Emotional abuse has been theorised to be particularly important for a negative cognitive style, “because the depressive cognitions (e.g., ‘‘You’re so stupid, you’ll never amount to anything’’) are directly supplied to the child by the abuser” (Gibb et al., 2001, p. 426), whereas physical and sexual abuse require children to make their own depressogenic interpretations. A growing volume of research also implicates neurobiological mechanisms involved in the relationship between childhood exposure to violence and psychopathology, including depression. Moffitt (2013) identifies various different pathways whereby such stressful events may “get under the skin”, including altered inflammatory reactions, alterations in brain areas associated with mood regulation, telomere erosion, epigenetic methylation, and altered gene expression (see also, Nemeroff (2016)).

Of course, just because the current study found that maltreatment predicted depression only for girls (and only with respect to emotional abuse and exposure to domestic violence) does not mean that maltreatment of boys and all types of maltreatment are not important. Maltreatment is a human rights issue, as well as a health issue, and has been linked to various other adverse outcomes, for males as well as females, including difficulties in educational achievement, childhood behaviour problems, post-traumatic stress disorder, attempted suicide, alcohol problems, and later crime and violence (Gilbert et al., 2009). Within the Brazilian context, the negative consequences of maltreatment are particularly troubling given high rates of maltreatment, and relatively low government investment in maltreatment prevention programmes (Viola et al., 2016).

### 4.1 Strengths and limitations

A strength of this study was the use of a population-based, longitudinal study design with a large number of participants, and high follow-up rate up to age 18 years. Data were collected prospectively reducing selective recall bias and helping specify the temporal sequence of exposures and outcome. Furthermore, we included a wide range of confounding variables and adjusted for the co-occurrence of different types of maltreatment when estimating their individual effects. Information on exposures and confounding factors were obtained in early life and adolescence by trained interviewers. Another of the study's strengths was the use of a standardised and validated questionnaire to measure major depression.

Study limitations should also be considered. Of initial members of the cohort, 70.8% were included in the current analyses. There were some differences between those included in the analyses and those excluded; included participants were more likely to be female, and to have mothers with non-white skin colour, lower education, and lower family income. If effects of maltreatment varied according to these family characteristics, differential attrition could bias estimates of association in the current study. A second limitation was that an extensive and validated instrument on maltreatment was not used. More detailed information was not available, for example on the frequency, the severity, duration, chronicity of the exposures, and the victim-aggressor relationship. Maltreatment was assessed in a confidential self-report questionnaire, and although self-reported maltreatment is likely to involve fewer false-negatives than maltreatment data from other sources (Stoltenborgh et al., 2015), underreporting can still occur, particularly for sensitive events such as sexual assault. Because maltreatment was only measured once during the study, it was also not possible to investigate whether there were sensitive periods in which maltreatment has larger effects on depression. With regard to the assessment of depression, we used a dichotomous indicator of clinical depression, and it is possible that a different, dimensional measure might have revealed more subtle effects of maltreatment on depression for men that were not identified in this study. Another limitation was that, although numerous confounding factors were adjusted for in the current study, residual confounding cannot be ruled out, caused by unmeasured factors associated with both maltreatment and depression.

## 5 Conclusion

In conclusion, this study found that females exposed to emotional abuse and domestic violence were at increased risk for major depression in late adolescence. Prevention efforts need to protect children and adolescents, particularly girls exposed to multiple forms of maltreatment.
